# Multiomics integration for the function of bacterial outer membrane vesicles in the larval settlement of marine sponges

**DOI:** 10.3389/fmicb.2024.1268813

**Published:** 2024-02-26

**Authors:** Beibei Zhang, Chenzheng Jia, Mingyu Li, Kai Wang, Jun Chen, Jing Zhao

**Affiliations:** ^1^College of Ocean and Earth Sciences, Xiamen University, Xaimen, Fujian, China; ^2^Xiamen City Key Laboratory of Urban Sea Ecological Conservation and Restoration (USER), Xiamen University, Xiamen, Fujian, China

**Keywords:** symbiosis, larval settlement, outer membrane vesicles, multiomics, arginine, nitric oxide synthase

## Abstract

Bacterial outer membrane vesicles (OMVs) contain a variety of chemical compounds and play significant roles in maintaining symbiotic relationships in a changing ocean, but little is known about their function, particularly in sponge larval development. During the growth of sponge *Tedania* sp., OMVs from *Bacteroidetes* species significantly promoted larval settlement, and *Tenacibaculum mesophilum* SP-7-OMVs were selected as a representative strain for further investigation. According to OMVs metabolomics, larval settlement might be connected to organic acids and derivatives. The multiomics analysis of the *T. mesophilum* genome, SP-7-OMVs metabolome, and larval transcriptome revealed 47 shared KEGG pathways. Among the number of candidate metabolites, arginine was chosen for its greater ability to increase the settlement rate and its role as the principal substrate for nitric oxide (NO) synthesis of sponge larvae. In summary, these results demonstrated that sponge-associated bacteria might utilize OMVs and their cargo to support host development and make up for host metabolic pathway deficiencies. This study enhances our fundamental knowledge of OMVs in interactions between metazoan hosts and microorganisms that are crucial in the coevolution of marine ecosystems and the complex marine environment.

## 1 Introduction

Symbiosis, which is recognized as a central driver of evolution across the entire tree of life (Raina et al., 2018), can be observed nearly everywhere in the oceans (Sogin et al., 2020). The sponge holobiont, which is an example of the concept of nested ecosystems, is globally distributed in marine and freshwater habitats (Pita et al., 2016; Pronzato et al., 2017). The highly efficient filter-feeding capacity of sponges enables numerous microorganisms to colonize sponge tissues, supporting a high diversity of symbiotic relationships (Duriš et al., 2011). The sponge-associated microbial communities that coevolve with their hosts over time and space (Easson and Thacker, 2014) contribute to the overall development, health, and adaptive capacity of the sponge holobiont (Fieth et al., 2016; Thomas et al., 2016; Pita et al., 2018). Sponges have an ancient and conserved biphasic life cycle, beginning from a free-swimming phase to settlement and metamorphosis. The successful settlement and metamorphosis of larvae are largely linked to the distribution, growth, and survival of marine invertebrates (Treml et al., 2015). Increasing evidence has emphasized that microbes could regulate some of the crucial steps in animal development (Nyholm, 2020) regardless of whether bacterial transmission is categorized as vertical or horizontal (Carrier et al., 2022). Song et al. (2021) demonstrated that symbiotic bacteria synthesize the essential amino acid arginine to promote the settlement and metamorphosis of larvae of *Amphimedon queenslandica*. Nevertheless, the intricate mechanisms of interaction between microbes and sponge larvae while in a complex marine environment are rather unclear (Díez-Vives et al., 2022).

Understanding the substantial influence of the microbiome on the settlement of sponge larvae requires the identification of specific bacterial groups at critical growth stages. In our previous studies, members of the phylum Bacteroidetes associated with *Tedania* sp. increased sharply during the free-swimming period, which is critical to the settlement and metamorphosis of the larvae (Wu et al., 2018). Díez-Vives et al. (2022) showed that, when the larvae were released into the water column, they kept experiencing symbiont proliferation, which conferred larvae a certain level of ecological plasticity (Díez-Vives et al.,). According to reports, Bacteroidetes play important roles in a variety of marine environments and can degrade complex molecules found in the ocean's organic debris (Bauer et al., 2006). We also found that the outer membrane vesicles (OMVs) of Bacteroidetes species, which ranged in size from 50 to 200 nm, might contain effect components to facilitate the settlement of sponge larvae, significantly increasing the larval settling rate (Li et al., 2021). Despite being less explored, molecular cargo-carrying OMVs from marine bacteria were discovered to be a typical biological trait with an actual and unexpected function (Aschtgen et al., 2016). Sponges thrive and evolve in a sea of microorganisms, where bacteria produce chemical cues affecting the cellular behavior of nearby species. OMVs could shield effector molecules from the negative effects of nucleases and proteases, allowing them to be targeted to specific cells (Schatz and Vardi, 2018).

By facilitating the flow of information and energy, marine bacterial OMVs may become essential for maintaining the stability of marine ecosystems. The quorum sensing (QS) molecule CAI-1 could be sustained and transported over extensive distances when it was packaged in the OMVs of *Vibrio harveyi* (Li et al., 2016; Brameyer et al., 2018). *Vibrio fischeri* OMVs made significant contributions to the development of squid light organs (Aschtgen et al., 2016). Signal lipids found in OMVs of *Algoriphagus machipongonensis* induced the rosette formation of *Salpingoeca rosetta* (Woznica et al., 2016). However, due to numerous interactions occurring concurrently in the developmental process of marine sponges, it is still a challenge to investigate complex signals from related bacterial OMVs. It is also difficult to decipher effective chemical compounds and their roles without knowledge of the individual microorganisms. Based on our previous findings, it was determined that Bacteroidetes species-OMVs significantly facilitated sponge *Tedania* sp. larval settlement and metamorphosis. In this study, the metabolome of one representative bacterial OMVs was examined, and multiomics analyses of the bacterial genome and the transcriptome of sponge larvae were conjointly analyzed to identify crucial signals. As a result, potential metabolites were selected, and a series of experiments were conducted, including sponge larval settlement, liquid chromatography (LC)-mass spectrometry (MS), and real-time polymerase chain reaction (qPCR). The results suggested that OMVs could be one effective method for maintaining a symbiotic relationship in the sponge life cycle. This discovery advanced our knowledge of intricate symbioses and the shared metabolic processes that drove the coevolution of life.

## 2 Materials and methods

### 2.1 Bacterial cultivation, OMVs isolation, and purification

Six strains (accession number from MW24846 to MW24858) of Bacteroidetes were isolated from the larvae and adults of sponge *Tedania* sp. and were found to produce OMVs, including *Cryomorphaceae bacterium* SW-4, *Formosa* sp. SW-1, *Salegentibacter* sp. SP-1, *Sunxiuqinia dokdonensis* SP-8, *Muricauda aquimarina* SP-4, and *Tenacibaculum mesophilum* SP-7 (Li et al., 2021). They were cultivated aerobically in marine 2216E broth at 28°C. The OMVs were isolated from 500 ml bacterial culture (OD_600_ = ~1.0) in accordance with a modified procedure. After all bacterial cells were removed via centrifugation at 2,627 × g for 40 min, the OMVs were separated from other extracellular products by ultracentrifugation at 100,000 × g for 1 h at 4°C (41 Ti Rotor, Beckman Coulter, California, USA). The resulting pellets were washed and re-suspended in 10 mM phosphate-buffered saline (PBS; Solarbio, Beijing, China.) (Brown et al., 2015). The amount of OMVs was determined corresponding to the protein content by using the Modified Bradford Protein Assay Kit (Sangon Biotech, Shanghai, China.). The bacteria and OMVs were observed by scanning electron microscopy (SEM) and transmission electron microscopy (TEM), respectively.

### 2.2 Marine sponge larval settlement assay

Free-living larvae of marine sponge *Tedania* sp. were prepared by a tuck net with a mesh screen (150 μm) (Li et al., 2021). The effects of six Bacteroidetes strains and their OMVs were assessed on larval settlement and metamorphosis in sterile six-well plates (NEST Biotechnology Co., Ltd., Wuxi, China). The larvae were incubated in the control groups of nature seawater (NSW) and filtered seawater (FSW) with 0.22 μm pore-sized polyvinylidene fluoride membrane filters (Millipore Co., Ltd, Darmstadt, Germany). The NSW group was used to demonstrate the health of the collected sponge larvae and their ability to live in a natural setting. To evaluate the effect of bacteria and OMVs addition on larval settlement, FSW was used as the basic experiment seawater without taking other biological components into account. All bacteria were quantified up to 10^3^ cfu/ml and added into FSW. According to references reported, the method of quantitative protein was an approved way for quantifying OMVs (Klimentová and Stulík, 2015). OMV proteins were extracted using a Bacterial Protein Extraction Kit (Sangon Biotech) and quantified by using the Bradford Protein Quantification Kit (Vazyme Biotech Co., Ltd, Jiangsu, China). Based on previous experimental results, most bacterial species-OMVs showed the highest settlement rates at a medium concentration (~100 ng/ml protein) (Li et al., 2021). Therefore, for each OMV experiment, a protein concentration of approximately 100 ng/ml was chosen, and the corresponding OMVs were incubated in 8 ml FSW. One parallel group contained ≥30 sponge larvae and three parallel plates were set for each trial. After 8–90 h, the amount of settled larvae was counted to evaluate the ability of larval settlement. The effects of bacteria and vesicles on settlement rates were computed independently by SPSS (a one-way ANOVA analysis), and descriptive analyses, homogeneous subset tests, and tests of homogeneity of variances were carried out. Afterward, the OMVs and sponge cell suspensions after incubation were observed by SEM.

### 2.3 Multiomics analysis of the bacterial genome, larval transcriptome, and OMVs metabolome

#### 2.3.1 The *T. mesophilum* genome analysis

To ascertain the bacterial source of OMVs-metabolites, the analysis of the genome sequence was required. Genomic DNA was extracted using HiPure Bacterial DNA Kits (Magen, Guangzhou, China) according to the manufacturer's instructions. The DNA quality was detected using Qubit (Thermo Fisher Scientific, Waltham, MA) and NanoDrop (Thermo Fisher Scientific, Waltham, MA), respectively. *T. mesophilum* SP-7 genomic DNA was sequenced on the Illumina/PacBio sequencing platform (Genedenovo Biotechnology Co., Ltd, Guangzhou, China). Continuous long reads were attained from single-molecule real-time sequencing runs and adopted for ***de novo*** assembly by using Falcon (version 0.3.0). Raw data from the Illumina platform were filtered using FASTP (version 0.20.0) (Cleaning Reads: 14521406), and then, the final genome sequences were determined using Pilon (version 1.23). The genes were annotated by aligning them with genes deposited in diverse protein databases, particularly the Kyoto Encyclopedia of Genes and Genomes (KEGG).

#### 2.3.2 The *T. mesophilum* SP-7-OMVs metabolome analysis

The metabolites from *T. mesophilum* SP-7-OMVs were extracted, and **six** parallel samples (labeled EV-1-1, EV-1-2, EV-1-3, EV-2-1, EV-2-2, and EV-2-3) were analyzed by ultrahigh-performance LC (UHPLC) coupled with Q Exactive (QE) Orbitrap/MS utilizing a nontargeting approach (Guangzhou Genedenovo Biotechnology Co., Ltd.). LC-tandem MS (MS/MS) analyses were performed by using a UHPLC system (1290, Agilent Technologies) with an Acquity HSS T3 column (2.1 × 100 mm; 1.7 μm) coupled with QE (Orbitrap MS, Thermo Fisher Scientific). The acquisition software (Xcalibur 4.0.27, Thermo Fisher Scientific) continuously evaluated the full-scan survey MS data as it collected and triggered the acquisition of MS/MS spectra depending on the preselected criteria. MS raw data (.raw) files were converted into the mzML format using ProteoWizard and processed via R package XCMS (version 3.2). OSI-SMMS (version 1.0, Dalian Chem Data Solution Information Technology Co., Ltd) was used for peak annotation after data processing with the in-house MS/MS database. Metabolites were matched with MS2 (the second-order mass spectrum), in which these metabolites could be defined as a “superclass”. Metabolites were mapped to the KEGG metabolic pathways for further shared pathway analysis (Supplementary material 1.1).

#### 2.3.3 Shared KEGG pathway analysis and multiomics integration

To screen potential OMVs signal data for, the *T. mesophilum* genome and the larval transcriptome during the development stage (Supplementary material 1.2) were connected to bacterial OMVs' metabolome. As a result, the microbe-OMVs-sponge shared KEGG pathway was analyzed, and a correlation schema was constructed. In this study, the competent larva and the post-larva were used as samples for two crucial developmental stages, during which changes in transcript levels were closely correlated with the growth and maturation of the sponge larvae. As a consequence, the KEGG enrichment pathway, which incorporates DEGs, was employed. First, KEGG pathways focusing on the animal development process were enriched in DEGs from the larval transcriptome of *Tedania* sp. Second, all known metabolites from SP-7-OMVs were annotated in KEGG pathways (Supplementary material 1.1). Third, all known genes from the *T. mesophilum* genome were assigned to KEGG pathways. Using the shared KEGG analysis, pathways associated with key metabolites were discovered. Thus, the metabolites of MS2 were selectively analyzed. Then, the literature was examined for topics pertaining to marine invertebrate development. After the candidate compounds had been identified, the *T. mesophilum* genome was used to identify the main enzyme genes responsible for manufacturing the potential molecules, and the transcriptomes of sponge larvae were thoroughly examined to look for DEGs associated with the potential metabolites. By using the optimal acting concentration of various metabolites, the effective molecules on sponge larval settlement could be finally determined.

### 2.4 Quantification and functional verification of candidate metabolites from SP-7-OMVs

The salt was initially removed from OMVs samples using a Millipore volume with a 100-kDa filter membrane. Four volumes of cold methanol were added into the samples, broken for 12 min by ultrasonication using a noise isolating chamber (Scientz Biotechnology Co., Ltd, Ningbo, China), and kept for 1 h at −20°C for albumen precipitation. The samples were subsequently centrifuged at 12,000 rpm for 15 min at 4°C. Using a Termovap Sample Concentrator (N-EVAP), the supernatant was reduced to the smallest possible volume before being dried and re-dissolved in a minimal amount of water. The flow rate for LC-MS was 3 μl/min, and a gradient of acetonitrile (ACN) was used to elute the analytes. The analytes were initially eluted at 1% ACN for 3 min, followed by 60% for 10 min, 95% for 12 min, and then back to the initial 1% ACN for 20 s. Re-equilibration took place for 20 min. Data were manually annotated using Xcalibur Qual Browser software (Thermo Fisher Scientific).

### 2.5 qPCR analysis of the nitric oxide synthase (*NOS*) expression

RNA was extracted from a variety of materials using the miRNeasy Mini Kit. The samples used in the *NOS* gene quantification experiment were an embryo, competent larva, post-larva, and “OMVs+post-larva”. The embryo and competent larvae were selected for collection from NSW due to the particularity of the embryo in the adult sponge and its special time node when floating larvae were just being released from the body. Following a roughly 24 h co-incubation in FSW, the post-larva and “OMVs+post-larva” were collected for qRT-PCR analysis, which were consistent with transcriptome. Among them, the competent larvae act as the blank control. RNA was reverse-transcribed into cDNA by the PrimeScript^TM^ RT reagent kit with a gDNA Eraser (Takara Co., Ltd.). qPCR was performed using a QuantStudio 6 Flex instrument (Life Technologies) via TB Green^®^Premix Ex Taq™II (Tli RNaseH Plus) (Takara Co., Ltd.). Specific primers were designed for the *NOS* and succinate dehydrogenase complex, subunit A (*SDHA*) genes in line with the transcriptome of *Tedania* sp. larvae. The quantity of *NOS* transcripts throughout normal development was evaluated using qPCR with the following oligonucleotide primer set: forward 5′- AATTTGCTCAAGCCCATTGC-3′ and reverse 5′-GAGGGACAATCCACACCCAG-3′. The transcription level was normalized using the reference gene SDHA (Gardères et al., 2014; Song et al., 2021) to obtain the relative gene expression values for *Tedania* sp. The oligonucleotide primer set for *SDHA* was as follows: forward 5′- CTCTTTCCCACCCGATCTCAC-3′ and reverse 5′- GGGCATTCCGTAATTCTCAAGC-3′. The relative level of the *NOS* gene expression was calculated using the 2 ^−Δ*ΔCt*^ method (Livak and Schmittgen, 2001), and three replicates were performed for each treatment.

### 2.6 Statistical analyses

All data were expressed as the means ± standard deviations from at least three sets of independent experiments. Both GraphPad Prism 9.0 software (GraphPad Software, Inc., San Diego, California, USA) and Microsoft Excel (Microsoft Corporation, Seattle, Washington, USA) were used to analyze all data. Using a one-way ANOVA and *t*-tests, significant variations in the sample characteristics were assessed. The results were displayed using online tools (HiPlot, Figdraw, and Vecteezy), as shown in the Figures 2, 5.

## 3 Results

### 3.1 Effect of bacteria and bacteria-OMVs on sponge larval settlement

**Figure 1:**
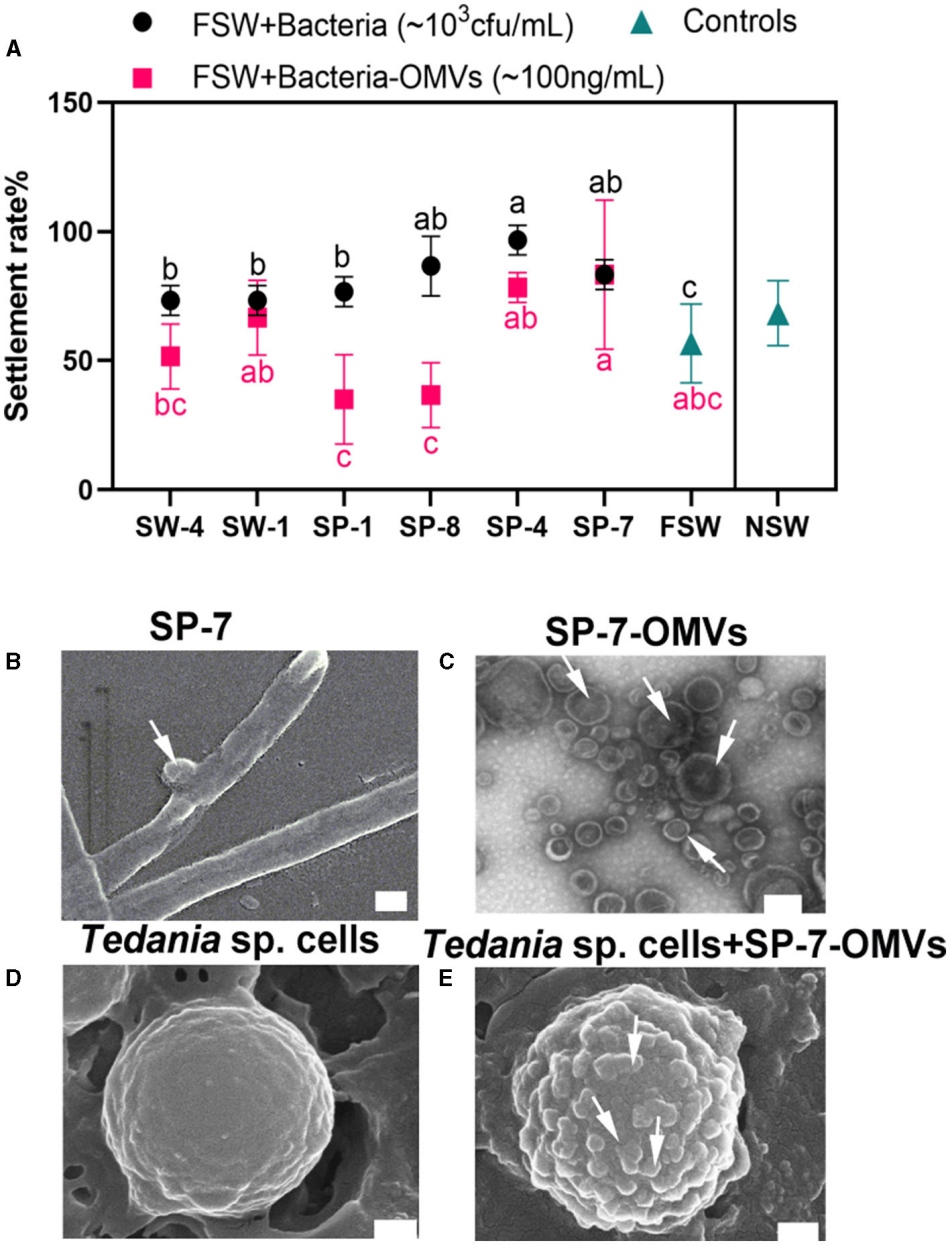
Effect of Bacteroidetes species and bacteria-OMVs on the settlement of sponge larvae. **(A)** Effect of bacteria and bacteria-OMVs on sponge larval settlement. Bars show the mean ± SD from three prepared independent samples. The black solid circles represent each experimental group, where bacteria were added to FSW, and the black “a-b-c” indicates whether the difference between the experimental group and the FSW group was significant. Rosy red squares represent the individual experimental groups with the addition of bacterial vesicles to FSW, and rosy red “a-b-c” indicates whether the difference between the experimental group and the FSW group was significant. **(B)** SEM image of SP-7. **(C)** TEM image of SP-7-OMVs. **(D)** SEM image of sponge cells. **(E)** SEM image of SP-7-OMVs and sponge cells. Scale bar: 200 nm. See also Supplementary Tables S1, S2.

To determine the effect of bacteria and bacteria-OMVs on sponge larval settlement, six Bacteroidetes species and their OMVs were examined. Without the addition of any other Bacteroidetes species and their OMVs, it was found that the sponge larval settling rates in NSW and FSW were approximately 68% and 56%, respectively, after approximately 24 h. All bacterial additions increased the larval settling rates in comparison to the FSW and NSW control groups. When strains SP-8, SP-4, and SP-7 were added to the FSW group, they exhibited considerable activities to promote larval settlement, even more than the NSW group (Figure 1A, Supplementary Tables S1, S2). Among them, OMVs from the strain SP-7 (83.33% settlement rates) showed a nearly equivalent effect to bacteria. OMVs produced by the strain SP-7 had an integral membrane structure and were measured at 50–200 nm (Figures 1B, C). When comparing the surface of the larval cells under SEM observation without OMVs addition (Figure 1D), more vesicular OMVs were enriched in the surface of sponge cells throughout the process of promoting sponge larval settlement (Figure 1E).

### 3.2 Metabolome analysis of *T. mesophilum* SP-7-OMVs

**Figure 2:**
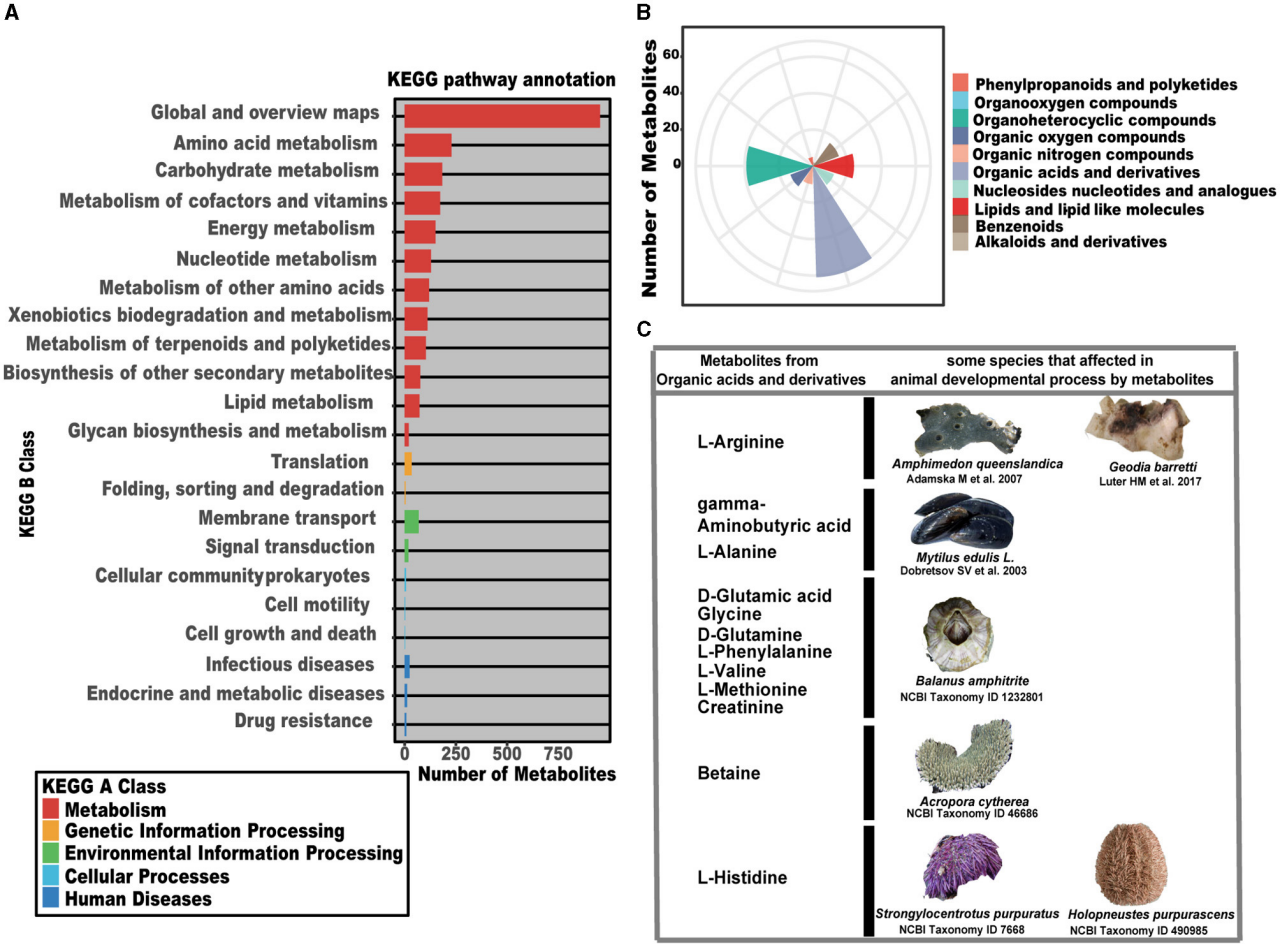
Metabolome analysis of *T. mesophilum* SP-7-OMVs. **(A)** The KEGG pathway annotation of metabolites. **(B)** The superclass of metabolites from MS2 and the annotated KEGG pathways. **(C)** Metabolites affect the animal development process in some species. See also Supplementary Figure S1, Supplementary Tables S3–S7.

Based on the fact that SP-7-OMVs significantly promoted sponge larval settlement, it was implied that OMVs could communicate with larval cells and the packaged signals could motivate larval development. To gain effective information, the non-targeted metabolome of *T. mesophilum* SP-7-OMVs was analyzed. The map of the total ion flow over time of six samples in negative and positive ion modes, which was used to determine their total ion count (TIC), was shown in Supplementary Figure S1, Supplementary Table S3. All known metabolites in SP-7-OMVs were annotated with the KEGG pathway, and 22 classes were traced to the KEGG B class (Figure 2A, Supplementary Tables S4, S5). Most of the metabolites were annotated to the kind of metabolism of the cluster KEGG A class. Among them, the categories belonging to global and overview maps, amino acid metabolism, and carbohydrate metabolism were the top three with the most amount of metabolites.

Superclass analysis was performed for 185 metabolites from MS2 that could be annotated into the KEGG pathway (Figure 2B, Supplementary Tables S6, S7). These organic compounds identified as organic acids and derivatives, organic nitrogen compounds, organic oxygen compounds, and organoheterocyclic compounds were predominate metabolites. Among them, the most abundant categories of metabolites were “organic acids and derivatives” (61 metabolites) and “organoheterocyclic compounds” (39 metabolites), many of which have been identified as potential effectors connected to animal development (Figures 2B, C). Arginine was reported to be involved in the growth and development of the marine sponges *A. queenslandica* (Song et al., 2021) and *Geodia barretti* (Hedner et al., 2008). Gamma-aminobutyric acid and L-alanine were shown to promote the development of the blue mussel *Mytilus edulis* L. (Dobretsov and Qian, 2003; Rivera-Ingraham et al., 2011). D-glutamic acid, glycine, D-glutamine, L-phenylalanine, L-valine, L-methionine, and creatine were confirmed to be connected with cypris larval growth and the development of the barnacle *Balanus amphitrite* (Tegtmeyer and Rittschof, 1988; Inoue et al., 2018; Labriere et al., 2021). Betaine was verified to induce the life cycle of the coral *Acropora cytherea* (Jorissen et al., 2021). L-histidine, as a signal molecule, was related to *Strongylocentrotus purpuratus* (Sutherby et al., 2012) and *Holopneustes purpurascens* (Swanson et al., 2004) (Figure 2C).

### 3.3 Multiomics analysis

**Figure 3:**
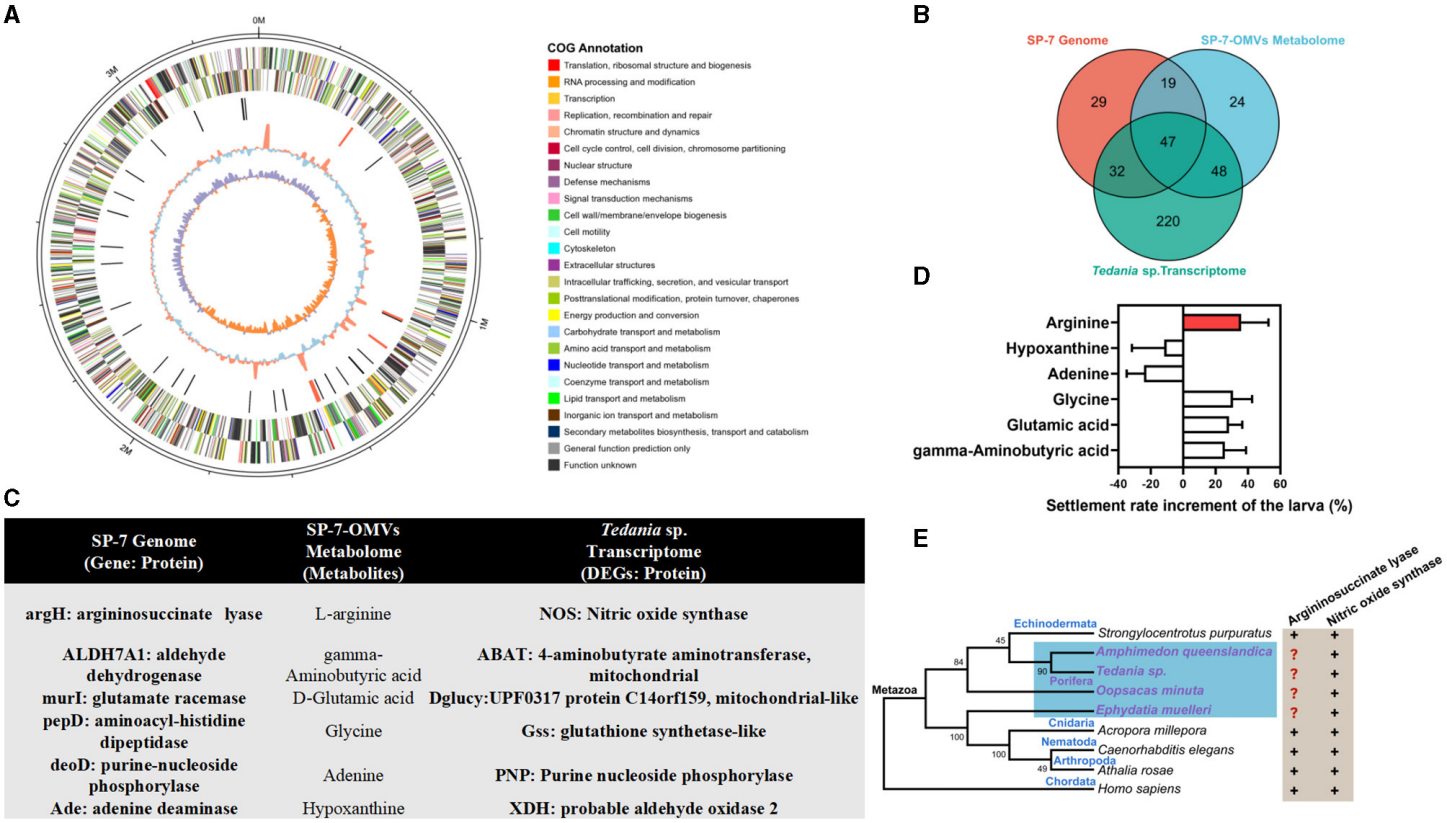
Multiomics analysis of the *T. mesophilum* genome, SP-7-OMVs metabolome, and *Tedania* sp. larval transcriptome. **(A)** The circos diagram of the *T. mesophilum* genome. **(B)** A Venn diagram of the number of shared KEGG pathways in multiomics. **(C)** Upstream and downstream information of candidate key metabolites in multiomics. **(D)** Effects of different metabolites on sponge larval settlement increase. **(E)** The relationship between life evolution and the key gene that encodes argininosuccinate lyase and NOS. Neighbor-joining method and 1,000 bootstrap replications. See also Supplementary Figures S2–S7, Supplementary Tables S8–S14.

The multiomics analysis, which included the bacterial genome and the transcriptome of the larval host, validated the true bacterial origin of these metabolites and their possible impact on the larval host. The strain *T. mesophilum* genome, which had 3,360,630 bp in total length and 31.76% in GC content, was sequenced to confirm the bacterial origin of the metabolites found in OMVs (Figure 3A). A total of 7834 DEGs (Supplementary Table S8) were enriched into 347 KEGG pathways (Supplementary Table S9) from the *Tedania* sp. larval transcriptome. In total, 100 metabolites from “organic acids and derivatives” and “organoheterocyclic compounds” were enriched into 138 KEGG pathways (Supplementary Table S10) from the SP-7-OMVs metabolome. A total of 715 genes from the *T. mesophilum* genome were enriched into 127 KEGG pathways (Supplementary Table S11). These findings showed that the connectivity of 47 KEGG pathways (Supplementary Table S12) was shared in bacteria, OMVs, and sponge larvae (Figure 3B). Six metabolites were selected based on the above analysis, and a probable correlation with the larval settlement was previously described, including arginine, hypoxanthine, adenine, glycine, glutamic acid, and gamma-aminobutyric acid (Figure 3C, Supplementary Figures S2–S6).

Depending on the optimal concentration, arginine, glycine, glutamic acid, and gamma-aminobutyric acid could increase the sponge larval settlement rate by 35.84%, 30.83%, 28.33%, 25.83%, respectively (Figure 3D, Supplementary Figure S7, Supplementary Table S13). Among them, arginine exhibited the best performance and was targeted as one of the important metabolites for further verification of the interaction between bacterial OMVs and larvae. Afterward, the argininosuccinate lyases (*argH*) and nitric oxide synthases (*NOS*) that associated arginine as a substrate and that were involved in larval development were retrieved from the NCBI database. It was found that the majority of species contained both the *NOS* genes and *argH* genes, while sponges lacked the latter (Figure 3E, Supplementary Table S14). In other words, although sponges were unable to biosynthesize arginine, the *NOS* gene, which used arginine as a substrate, was fully expressed. Therefore, bacterial OMVs were reasonably speculated to transmit exogenous signaling molecules to sponge larvae, such as arginine.

**Figure 4:**
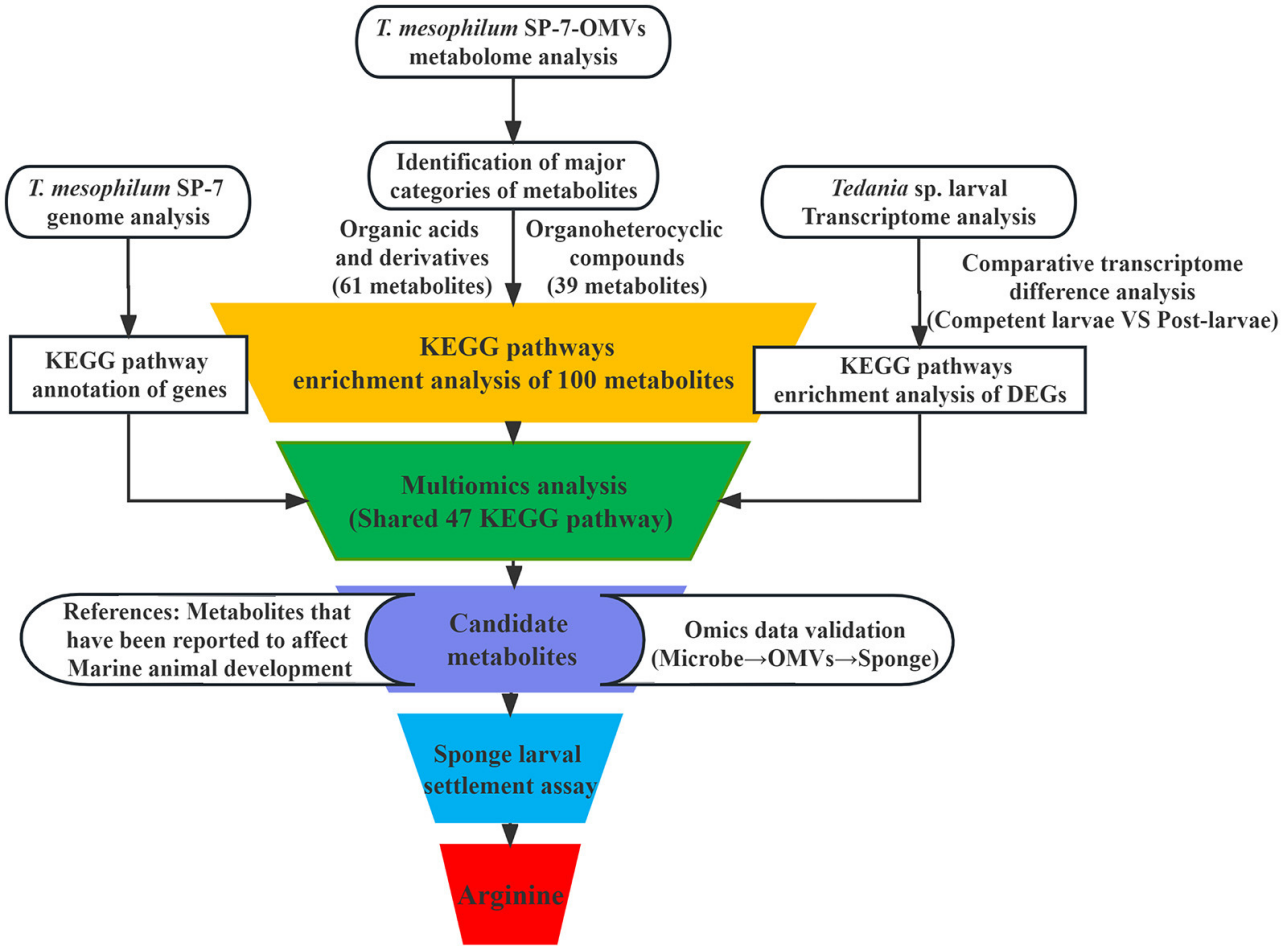
Multi-omics analysis workflow focusing on key metabolites.

The procedure was arranged for easier understanding, as shown in the illustration below (Figure 4). During this study, it made sense to use the three biologically relevant groups, including bacteria (*T. mesophilum*), bacterial OMVs, and sponge larvae (*Tedania* sp. larvae). The KEGG pathway analysis was performed by the *T. mesophilum* genome, SP-7-OMVs metabolome, and *Tedania* sp. larval transcriptome, respectively. Finally, a simple Venn diagram was used to locate the shared KEGG pathways.

### 3.4 A transport pattern for candidate metabolite mediated in the microbe-OMVs-sponge

**Figure 5:**
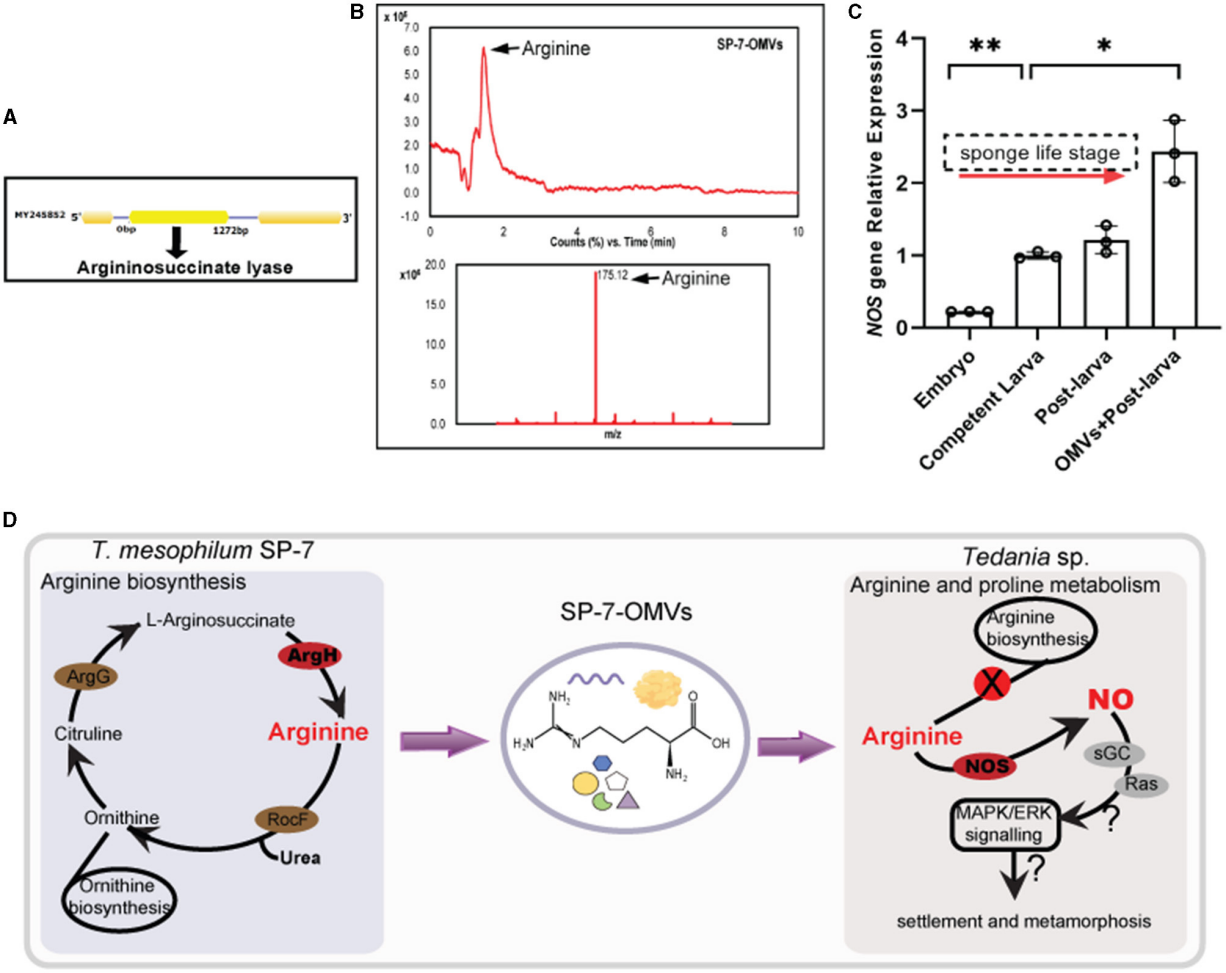
A joint analysis of arginine function in sponge larval settlement. **(A)** The structure of the *Arg*H gene that encodes argininosuccinate lyase. **(B)** An LC–MS analysis of arginine from SP-7-OMVs. Total ion current and m/z results are shown. **(C)** The expression of the *NOS* gene in four samples. Error bars show the mean ± SD for each treatment. Significance was determined by a two-sided unpaired Student's *t*-test (*p ≤ 0.05, **p ≤ 0.01). **(D)** The relationship model diagram of the *T. mesophilum* genome, SP-7-OMVs metabolome, and *Tedania* sp. transcriptome. See also Supplementary Tables S15, S16.

As a representative of many active substances, arginine was selected to testify to the function of bacterial OMVs by arginine quantification in OMVs, arginine's assay in larval settlement, and the related gene expression of larvae. First, the key enzyme argininosuccinate lyase (ArgH) located in the arginine biosynthesis pathway (ko00220) was detected with the gene size of 1,272 bp (Figure 5A) from the bacterial genome. Second, the arginine content was further measured by LC-MS according to the reference spectrum (m/z = 175.12) (Figure 5B, Supplementary Table S15). Compared with the standard, arginine from purified OMVs samples reached a high base peak intensity (RT = 1.45 min). The concentration of arginine in vesicles was estimated to be approximately 39.49 μg/L by HPLC, which was greater than the concentrations in seawater (approximately 5.58 μg/L) and seawater OMVs (approximately 0.07 μg/L). It implied that the presence of arginine in vesicles influenced sponge larval settlement more than NSW and FSW at the laboratory level. Finally, by qPCR test, the gene *NOS* expression increased with the transition of developmental stages in the sponge larval life cycle, from embryo to competent larva (swimming larvae) to post-larva (settled larvae) (Figure 5C, Supplementary Table S16). The post-larva incubated with SP-7-OMVs showed the highest relative expression of the *NOS* gene (*p* = 0.034).

## 4 Discussion

### 4.1 Bacterial OMVs function in symbiosis and marine ecosystems

Marine sponges arose more than 600 million years ago and could become promising models of animal-microbe symbioses (Li et al., 1998; Hentschel et al., 2012). Symbiotic bacteria coevolved with the sponge host, developing various strategies to grant hosts advantages or the “priority effect” in exchange for “refuge” from the sponge host (Björk et al., 2019). These bacteria aggregations could affect the larval growth process, even if it was unclear whether the proliferation of Bacteroidetes species in the late-larvae stage was the result of vertical transmission from parents' seed or horizontal transmission from surrounding seawater (Li et al., 2021). The OMVs from sponge-associated Bacteroidetes were favorably involved in larval settlement and metamorphosis according to our repeated tests, including the effect of different dose gradients of OMVs, the EM observation of larvae with or without OMVs addition, and the internalization of OMVs by larva using a confocal fluorescence microscope (Li et al., 2021). Similar roles of bacterial OMVs in larval development appeared in other invertebrate animals. Freckelton et al. (2017) found that large numbers of bacterial extracellular vesicles triggered the settlement of the larvae of *Hydroides elegans*. Guo et al. (2021) demonstrated that the enriched OMV fractions derived from *Pseudoalteromonas* sp. P1-9 induced high transformation rates of *Hydractinia echinata* larvae. The development of *Euprymna scolopes* could be stimulated by *Vibrio cholera* OMVs that contain a homolog of OmpU (Lynch et al., 2019). Prokaryotic organisms and animals have coevolved in the symbiotic connection for millions of years, and this interaction has been engineered in a variety of beneficial forms, such as the release of bacterial OMVs, to establish partners with the host (Hill and Round, 2021).

Despite the fact that free-arginine considerably increased sponge larval settlement in this study was consistent with earlier references (Song et al., 2021), the concentration was far greater than it was in seawater and bacteria OMVs. The availability of free-arginine in the dissolved form and the barrier effect of larval cells may limit the utilization of the bioactive molecule. OMVs are ubiquitous, lipid-bilayer-coated nanoscale membrane vesicles that enable active intercellular communications (Margolis and Sadovsky, 2019). Numerous OMVs from marine bacteria protect and condense biomaterials to create a stable transport channel for host-microbe cell interactions in a complicated marine environment with unlimited dilution and mobility (Zhao et al., 2022). These modularized OMVs from diverse bacteria with individualized components were delivered to eukaryotes and prokaryotes (Margolis and Sadovsky, 2019; Ñahui Palomino et al., 2021). For marine benthic species, ecological niche development and population viability depended on the successful larval settlement (Freckelton et al., 2017; Hamel et al., 2019). Therefore, bacterial OMVs could play an essential role in the flow of information and energy in marine ecosystems (Schatz and Vardi, 2018).

### 4.2 Bacterial OMVs effective information discovery

Once the biological role of OMVs in promoting larval settlement has been established, the identification of biomolecules becomes crucial for understanding the developmental and symbiotic mechanism of the host. A wide variety of metabolites, including steroid hormones, lipids, metabolic intermediates of nutrient anabolism, and monomers of major biopolymer classes, were present in SP-7-OMVs (< 1 kDa). These molecules may serve as a common language for host cells to learn (Ñahui Palomino et al., 2021). Several studies reported that the metabolites adenosine, acetylcholine, serotonin, palmitoleic acid, and others could induce larval settlement and metamorphosis of a variety of aquatic invertebrates, such as mussels, barnacle cyprids, and sea urchins (Kitamura, 1993; Faimali et al., 2003; Glebov et al., 2014; He et al., 2021). Numerous chemical cues carried by bacterial OMVs suggested their possible functions in interactions between bacteria and their animal host (Biller et al., 2014; Rudnicka et al., 2022). Furthermore, it was also verified that SP-7-OMVs, which carried signal molecules such as glycine, glutamic acid, and gamma-aminobutyric acid, could overcome cell barriers to aid in the growth and development of the host. The characteristic of bacterial OMVs effectors could give bacteria the inherent capacity to become an important component of sponge holobiont.

### 4.3 OMVs wrapped metabolite signals and promoted larval settlement

When bacterial genomics, the OMVs metabolome and the host transcriptome were combined with the shared KEGG pathway analysis to filter the impact molecules, a clear, complete information chain could be found present in the multiomics profile. There must be numerous bioactive molecules involved in the interaction between SP-7-OMVs and larvae that could be connected to larval settlement. Of them, arginine was selected since it clearly increased larval settlement rates. The arginine showed superior activity in enhancing sponge larval settlement, which was consistent with the impact of the exogenous NO donor (sodium nitroprusside and S-Nitroso-N-acetylpenicillamine) in *A. queenslandica* (Ueda et al., 2016). One crucial method for regulating larval settlement and metamorphosis in lower invertebrates is the NO pathway, which uses arginine as a substrate (Song et al., 2021). It has also been noted that the NO signal-induced larval settlement and metamorphosis occur via the MAPK/ERK signaling pathway (Ueda et al., 2016) in diverse marine invertebrates (Zhang et al., 2015; Yang et al., 2018; Song et al., 2021; Locascio et al., 2022). The NO signaling pathway involves the synthesis and utilization of arginine and includes the *argH* gene and the *NOS* gene. The *argH* genes, which are expressed in sponge-associated bacteria, are not encoded in the published genomic and transcriptomic data of the sponges (Srivastava et al., 2010). It was noteworthy that most invertebrates could synthesize arginine, whereas marine sponges were found to be defective in critical enzymes involved in arginine synthesis (Payne and Loomis, 2006). However, the associated bacteria with the sponge developed a “compensation mechanism” by which they could supply arginine to the host as a substrate for the *NOS* pathway (Song et al., 2021). Bacterial-OMVs could deliver arginine to cells and aid the growth and development of animal hosts (Figure 5D). Amino acid auxotrophy may be an evolutionarily optimal option for lowering biosynthetic burden (Mee et al., 2014), while related bacteria and their OMVs appear to provide metabolites as an ancient and alternative approach.

## 5 Conclusion

As an ancient sponge-bacteria holobiont, mutualism has been researched for over 100 years, but the mechanism by which the symbiont is implicated in larval development remains elusive. Studies on symbiosis and coevolution may raise a new viewpoint related to bacteria that produce vesicles preserving bacterial viability. Bacteria are unlikely to develop OMVs without a function because the formation and release of bacterial OMVs are energetically expensive (McMillan and Kuehn, 2021). The roles of OMVs produced by marine bacteria are not well understood, particularly in host settlement and metamorphosis. In this study, we discovered that Bacteroidetes associated with sponges could promote larval settlement by secreting numerous OMVs. Furthermore, we determined through multiomics integration that OMVs contained potential metabolites, mainly arginine, as necessary substrates to engage in the host's developmental process. In the marine environment and the nesting habitat, the OMVs of most bacteria serve as a conduit for communication across species because of their unique internal and surface characteristics. The temperature, pH, nutrient availability, and other physicochemical properties brought on by anthropogenic perturbations and global-scale climate change could have an impact on the expression of bacterial genes, their metabolism, their composition, and their adaptation to new environments (Hutchins and Fu, 2017). Therefore, environmental factors also inevitably affected the richness, composition, and even function of bacterial OMVs in the marine biosphere, which had a cascading effect on the composition of benthic communities and biogeochemical cycles.

## 6 Limitations of the study

In this study, the multiomics integration was used to demonstrate that bacteria SP-7-OMVs promoted sponge larval settlement by wrapping numerous metabolite signals. However, the NO pathway may not be the only way to regulate sponge larval settlement and arginine may be one of the effectors transported by bacterial OMVs. Larval settlement and metamorphosis of marine sponges are a complicated process. Future research would be focused on various metabolites and other compounds, such as microRNA, lipid, and protein, in order to comprehend the roles of bacterial OMVs.

## Data availability statement

The genome data were deposited at the National Center for Biotechnology Information (NCBI) database under accession number CP050861. The raw sequence data about the sponge larval transcriptome have been deposited in the Genome Sequence Archive (genomics, Proteomics & Bioinformatics, 2021) in National Genomics Data Center (Nucleic Acids Res, 2022), Beijing Institute of Genomics (BIG), Chinese Academy of Sciences, under the accession number CRA013645 (https://ngdc.cncb.ac.cn/gsa).

## Author contributions

BZ: Investigation, Methodology, Writing – original draft. CJ: Methodology, Writing – original draft, Conceptualization. ML: Investigation, Methodology, Writing – original draft. KW: Methodology, Writing – original draft, Investigation, Resources. JC: Resources, Writing – original draft. JZ: Resources, Conceptualization, Funding acquisition, Supervision, Writing – review & editing.
