# Supplementary material for: Multiomics integration for the function of bacterial outer membrane vesicles in the larval settlement of marine sponges
Source: Front Microbiol. 2024 Feb 26;15:1268813. doi: 10.3389/fmicb.2024.1268813 (PMC10925772; doi:10.3389/fmicb.2024.1268813)
Supplement: Supplementary file 2 [file Data_Sheet_1.docx]

Supplementary Material

# Supplementary Methods

1.1 **Analysis of metabolites annotation to KEGG pathway**

First, after normalizing the data using the total ion intensity, a data matrix of interesting features containing the retention time, *m/z* value, and normalized peak intensity was analyzed by Thermo Scientific™ Compound Discoverer™. Second, the metabolites were identified by the molecular formula prediction using molecular ion peaks and fragment ions, and were compared with the mzCloud (https://www.mzcloud.org/) and mzVault (https://mytracefinder.com/tag/mzvault/) and MassList (www.maldi-msi.org/mass) databases. Finally, the discovered metabolites were annotated using HMDB (http://www.hmdb.ca) and KEGG Compound database (http://www.kegg.jp/kegg/compound/), and the annotated metabolites were sequently mapped to KEGG Pathway database (http://www.kegg.jp/kegg/pathway.html) by C number. The procedure was available and had been applied in other investigations (Xu et al., 2021; Yu et al., 2022).

1. Xu J,Yu X,Ye H, et al. Comparative Metabolomics and Proteomics Reveal Vibrio parahaemolyticus Targets Hypoxia-Related Signaling Pathways of Takifugu obscurus. Front Immunol. 2021;12:825358. doi:10.3389/fimmu.2021.825358.
2. Yu L,Yang Y,Xiong D, et al. Phosphoproteomic and Metabolomic Profiling Uncovers the Roles of CcPmk1 in the Pathogenicity of Cytospora chrysosperma. Microbiol Spectr. 2022;10(4):e0017622. doi:10.1128/spectrum.00176-22.

### 1.2 *Tedania* sp. larval transcriptome analysis

### Total RNA of *Tedania* sp. larvae was extracted by using an miRNeasy Mini Kit (Qingen, Germany). Then, the enriched mRNA was fragmented into short fragments by using a fragmentation buffer and reverse transcribed into cDNA with random primers. The cDNA libraries were sequenced on the Illumina sequencing platform by Guangzhou Genedenovo Biotechnology Co., Ltd. Raw data from the Illumina platform were filtered using FASTP (version 0.18.0) (Cleaning Reads: 38677916- 45898162). The samples of planktonic and settlement larvae (labelled FY-1, FY-2, FY-3, BT-1, BT-2, and BT-3) were compared by analyzing differences in gene expression change. The unigene expression was calculated and normalized to reads per kilobase per million reads. The unigenes were annotated by alignment with those deposited in diverse protein databases. RNA differential expression analysis was performed between two different groups by using DESeq2 software. The KEGG pathway enrichment analysis identified significantly enriched metabolic or signal transduction pathways in differentially expressed genes (DEGs) by comparing two samples.

# Supplementary Figures

Figure S1 Metabolome analysis for *T. mesophilum* SP-7-OMVs. (A) and (B) Total ion chromatogram (TIC) diagram of each sample in negative and positive ion mode.

Figure S2 The KEGG pathway (ko00220), which arginine was located, including *argH* and *NOS*.

Figure S3 The KEGG pathway (ko00330), which gamma-Aminobutyric acid was located, including *ALDH7A1* and *ABAT*.

Figure S4 The KEGG pathway (ko00471), which D-Glutamic acid was located, including *murI* and *Dglucy*.

Figure S5 The KEGG pathway (ko00260), which Glycine was located, including *pepD* and *Gss*.

Figure S6 The KEGG pathway (ko00230), which adenine and hypoxanthine were located, including *deoD*, *PNP*, *Ade* and *XDH*.

Figure S7 Effects of different concentrations of glycine (A), glutamic acid (B), gamma aminobutyric acid (C), adenine (D), hypoxanthine (E) and arginine (F) on larval settlement within a limited observation time, related to Fig 3(C).


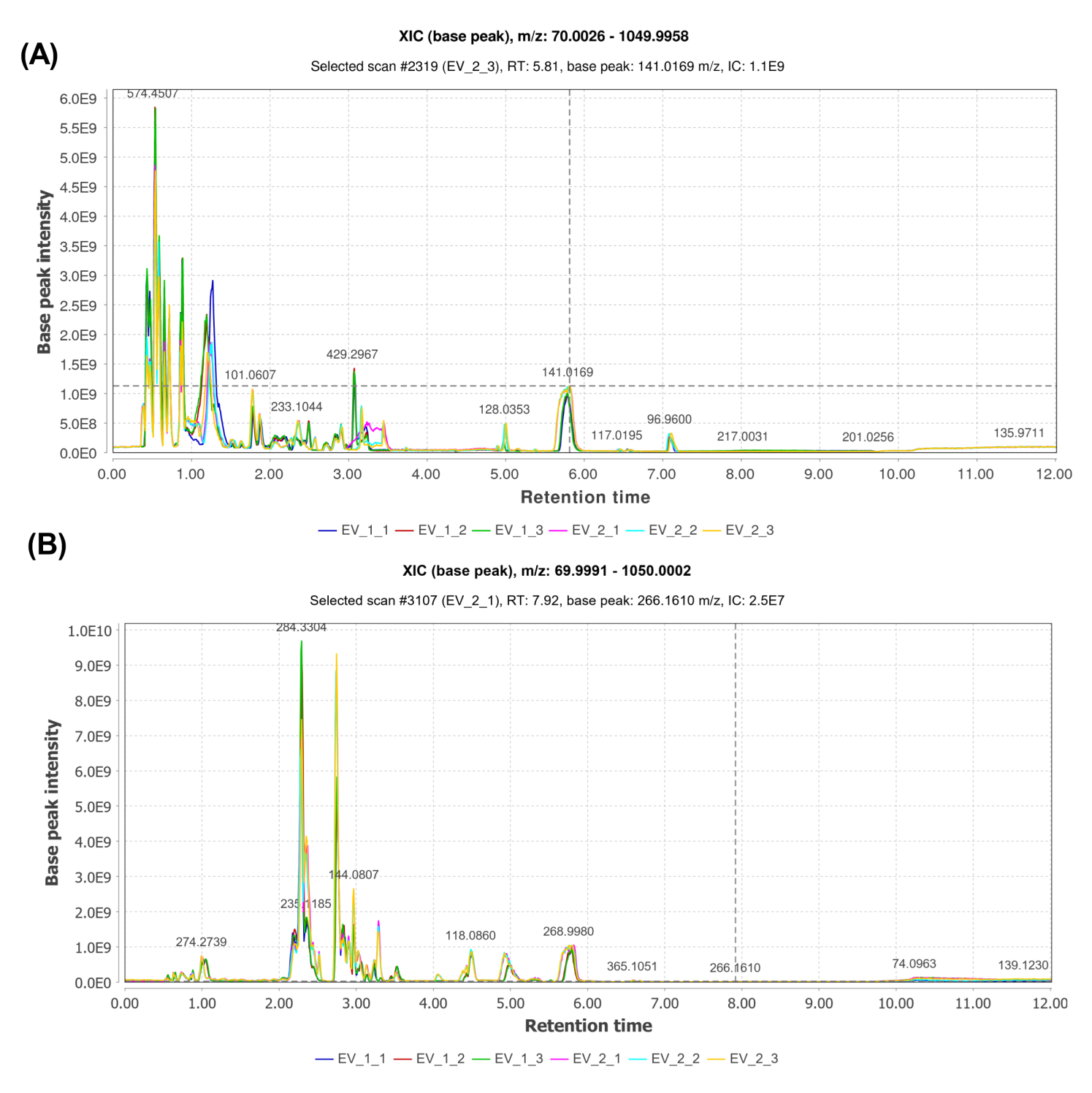


**Supplementary Figure 1.** Metabolome analysis for *T. mesophilum* SP-7-OMVs. (A) and (B) Total ion chromatogram (TIC) diagram of each sample in negative and positive ion mode.


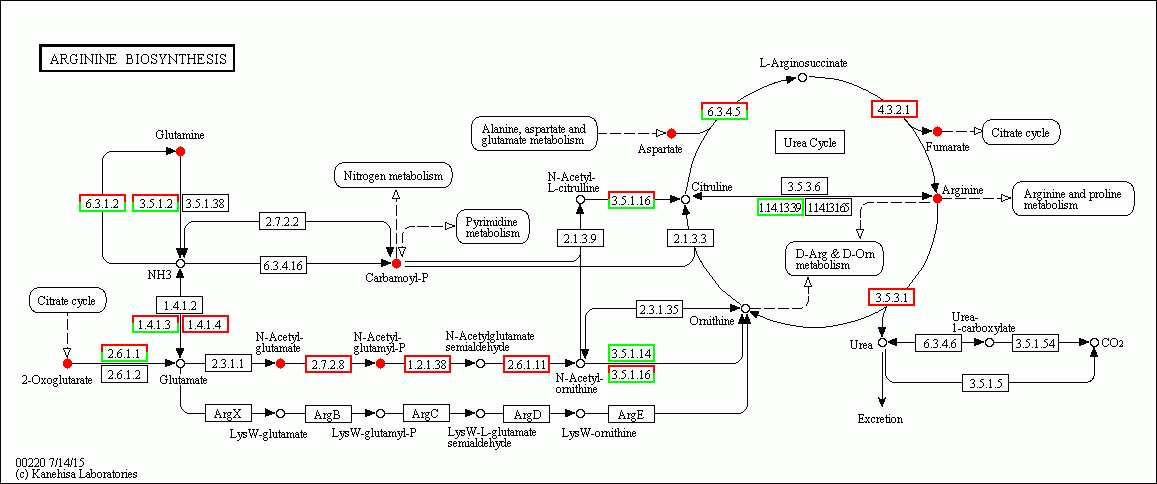


**Supplementary Figure 2.** The KEGG pathway (ko00220), which arginine was located, including *argH* and *NOS*.


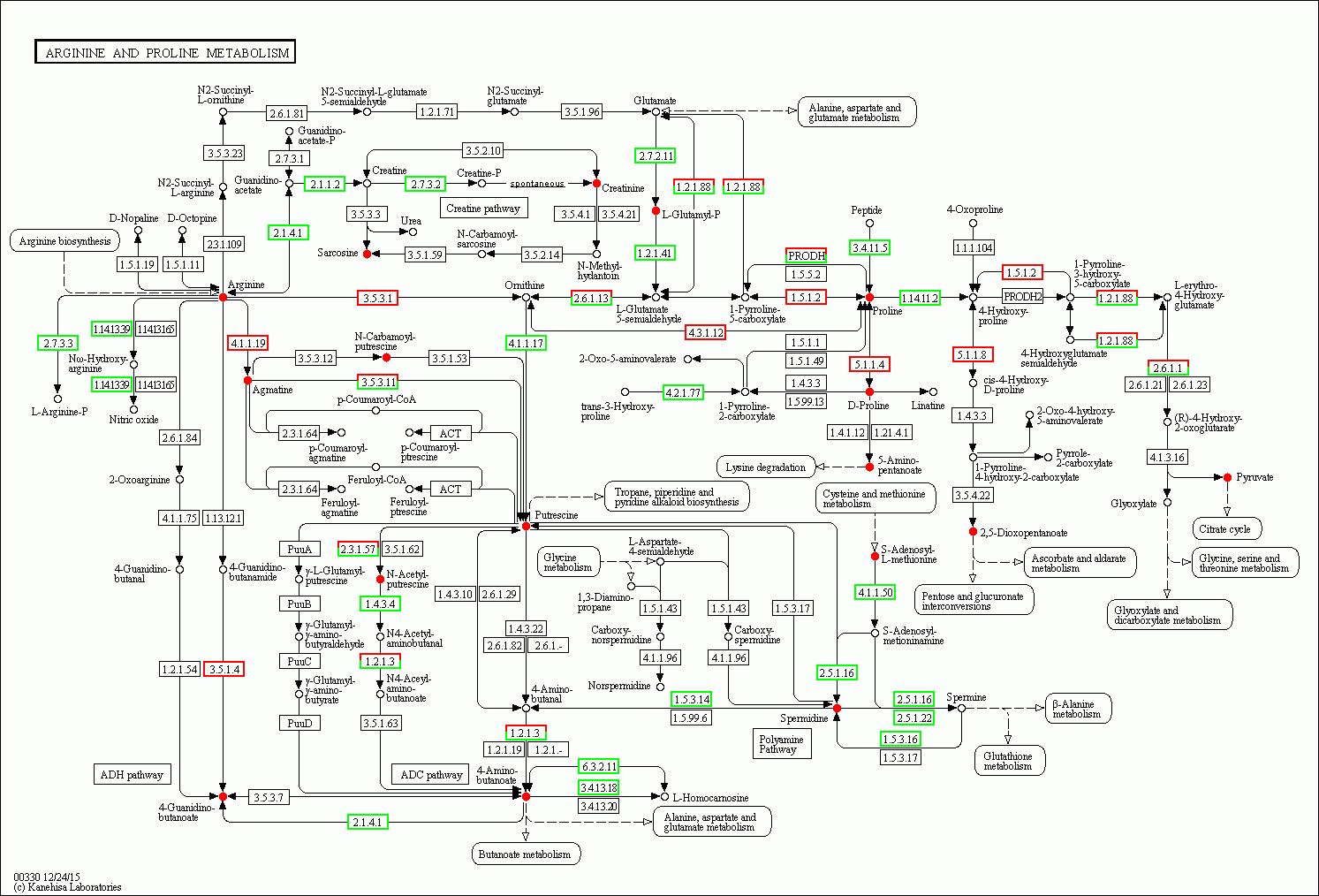


**Supplementary Figure 3.** The KEGG pathway (ko00330), which gamma-Aminobutyric acid was located, including *ALDH7A1* and *ABAT*.


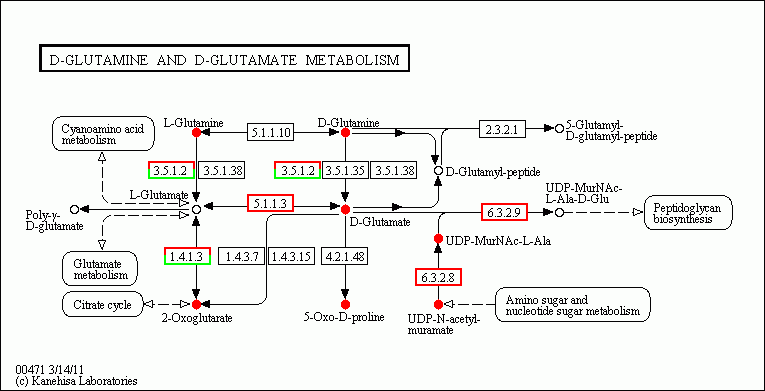


**Supplementary Figure 4.** The KEGG pathway (ko00471), which D-Glutamic acid was located, including *murI* and *Dglucy*.


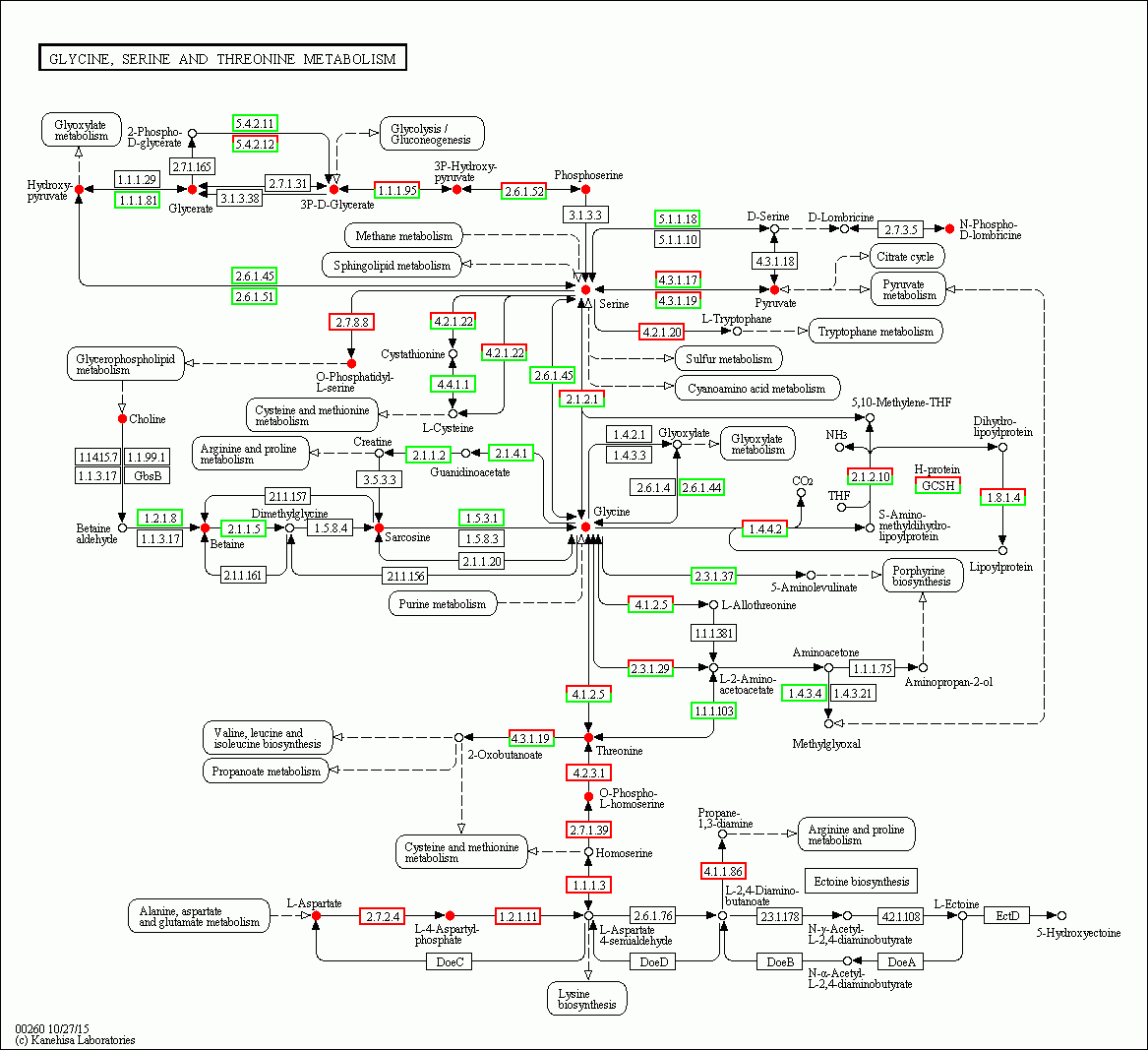


**Supplementary Figure 5.** The KEGG pathway (ko00260), which Glycine was located, including *pepD* and *Gss*.


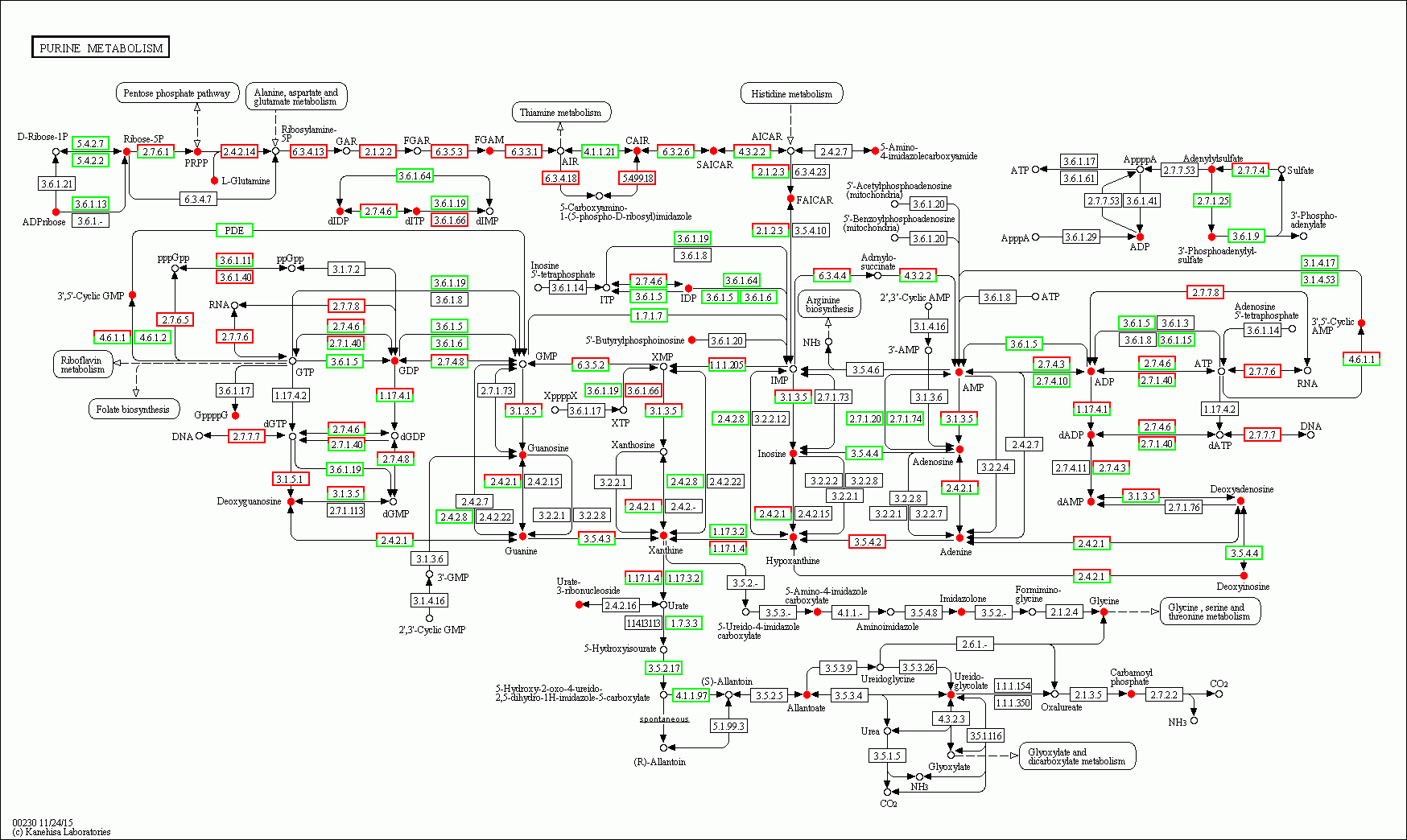


**Supplementary Figure 6.** The KEGG pathway (ko00230), which adenine and hypoxanthine were located, including *deoD*, *PNP*, *Ade* and *XDH*.

Figure S2-S6, red dots stand for metabolites, red boxes for genes exclusive to the genome, green boxes for transcriptome-DEGs, and half red and half green boxes for genes shared between the transcriptome and genome (i.e., the same KO number).


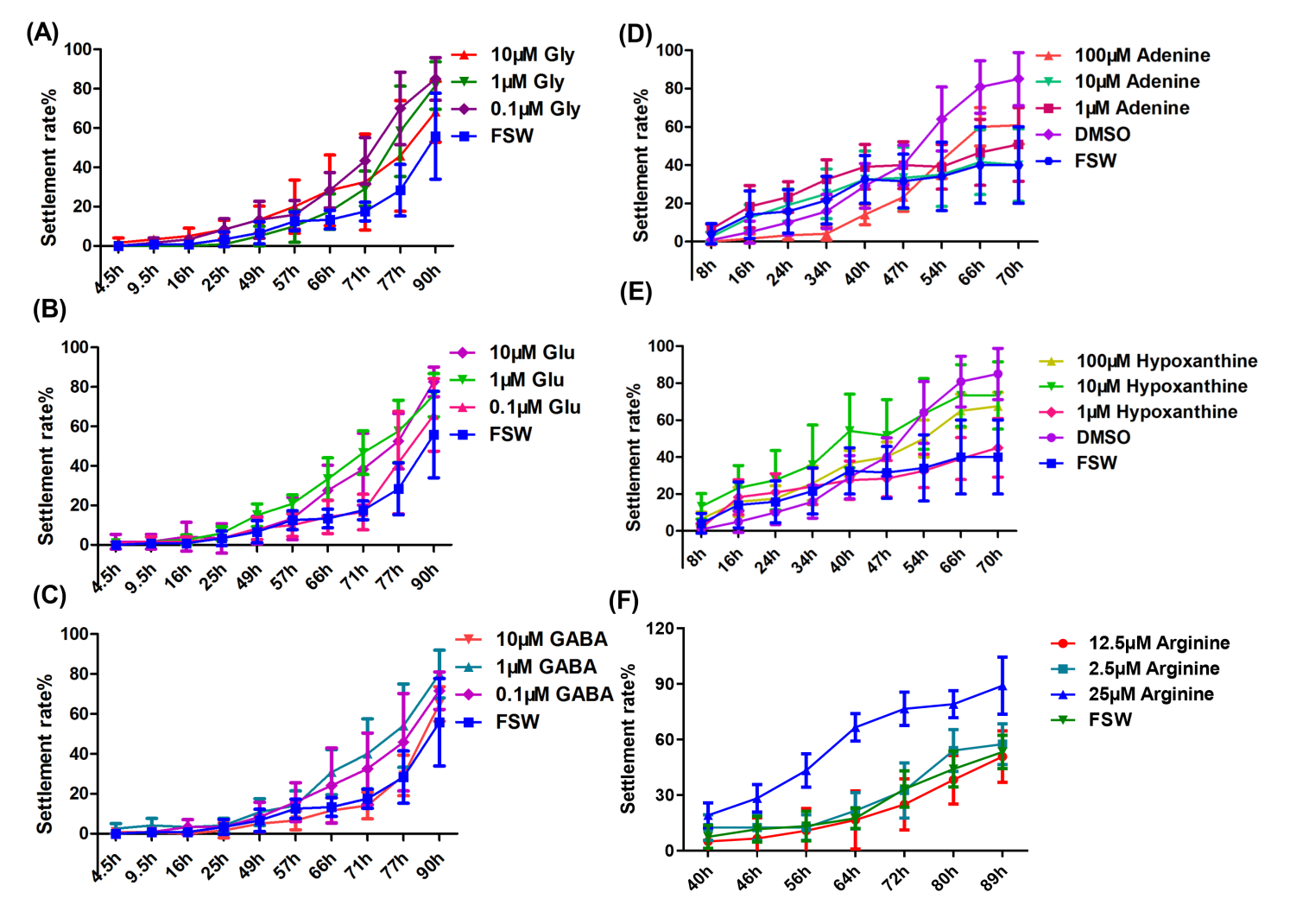


**Supplementary Figure 7.** Effects of different concentrations of glycine (A), glutamic acid (B), gamma aminobutyric acid (C), adenine (D), hypoxanthine (E) and arginine (F) on larval settlement within a limited observation time, related to Fig 3(D).
